# Castle man disease: a case report and review of the literature

**DOI:** 10.11604/pamj.2014.19.130.5045

**Published:** 2014-10-06

**Authors:** Mounir Kettani, Nabil Touiheme, Hicham Attifi, Mounir Hmidi, Ali Boukhari, Mohamed Zalagh, Abdelhamid Messary

**Affiliations:** 1 ORL and Cervical Facial Unit, Hôpital Militaire Moulay Ismail, Meknes, Morocco

**Keywords:** Angiofollicularlymphoid hyperplasia, cervical tumefaction, angiomatous, lymphoid, hamartoma, lymphoma

## Abstract

The Castleman disease (CD) is a rare disease of unknown etiology, characterized histologically by angiofollicular lymphoid hyperplasia. It comes in two forms, unicentric and multicentric. We report a case of Castleman disease in a 58 year old man, who had consulted for chronic cervical lymphadenopathy. This case was a multicenteric and rapidly fatal despite aggressive treatment with corticosteroids, and chemotherapy

## Introduction

The angiofollicular hyperplasia or Castleman disease (CD) was individualized for the first time by Castleman in 1956. It is a benign atypical lympho proliferation of unknown etiology [1], considered a prelymphomatose state. It comes in two forms, unicentric and multicentric, which are different in their clinical presentation and evolution [1]. In this work,from an observation of a patient with systemic CD, we propose the pathogenesis of multicenteric CD, it's clinical features, histopathology, treatment modalities, evolutionary aspects, and prognosis.

## Patient and observation

**Figure 1:**
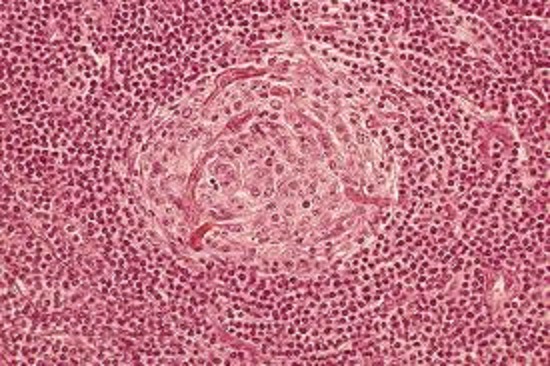
Castleman disease: lymphoid depletion centrofollicular. Thickening of the mantle zone having an aspect of “onion bulb”

**Figure 2:**
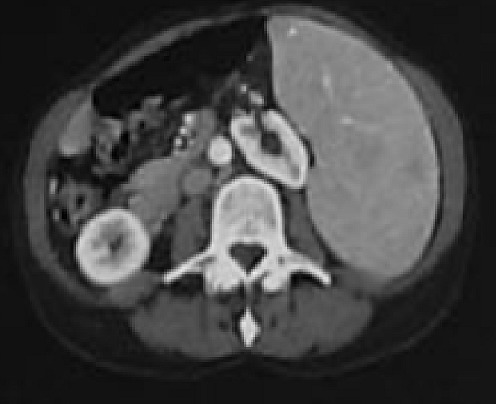
Castleman disease: splenomegaly

This was a case of 58 years old patient without significant pathological history, who was admitted in 2009 for exploration of cervical lymphadenopathy associated with impaired general condition lasting for more than a month. Clinical examination revealed an afebrile patient in poor condition; (ECOG = 2) with the presence of cervical and axillary lymphadenopathy, 2 to 3 cm in diameter, mobile, firm and painless. Splenomegaly reaching the left iliac fossa and, vascular purpura situated on inclined regions. The rest of the ENT examination was unremarkable, and the nasopharynx was free (no abnormality). Laboratory tests showed normocytic normochromic aplastic anemia with hemoglobin level 9 g/100 ml, sedimentation rate accelerated to 130 minutes in the 1st H, and the first H-HyperGamma polyclonal globulin level 26.1 g / l. Liver function checked by the coagulation test and LDH were normal. The HIV serology was negative. Lymph node biopsy with histopathologic examination concluded angiomatous lymphoid hamartoma (Figure 1). The thoracoabdominal CT scan revealed the presence of retro peritoneal lymph nodes and bilateral iliac small associated with splenomegaly (Figure 2), and a blade of ascites. An investigation for autoimmune disease showed the presence of antinuclear antibodies with speckled pattern and titre of 1/100. The patient was diagnosis for angiomatous lymphoid hamartoma in the multicentric form, was and treated with 3 courses COP (cyclophosphamide, vincristine, endoxan)at the rate of one cure per month. The evolution was marked by the complete regression of cervical lymphadenopathy. The patient died three months after the end of treatment in an array of oligoanuric renal failure.

## Discussion

The hamartoma angiomatous lymphoid or hyperplasia angiofollicular is a tumor of lymphoid tissue described for the first time in 1956 as a pseudo-tumor benign mediastinal. It often develops in a nodal structure, more rarely in the connective tissue. It is relatively rare since there are only400 observations in the literature [1, 2]. The diagnosis of CD is histological and it manifests in two forms;the most common vasculohyaline form (85%), characterized by the presence of lymphoid follicles abnormal, increased vascularity of the interfollicular area, small germinal center, and hyalinization of the vessels, and less frequent plasma cell shape form (15%), characterized by the presence of large follicles with germinal center hyperplasia, an almost complete absence of hyalinization, and a massive accumulation of plasma cells in the interfollicular area [1]. It also describes a mixed form, found occasionally as the separation between the two previous forms is not always easy [1, 3].

There areseveral assumptions proposed for the etiology of CD. The metaplastic or immune theory explains that disease caused by lymphoid hyperplasia reaction to various stimuli (trauma, inflammation) and it seems to be the most plausible in the forms with cervical facial locations [2]. Dysembryologique a theory was suggested. Other hypotheses have also been suggested involving the existence of prior immunosuppression with superimposed role of viruses, including HIV (human immunodeficiency virus), EBV (Epstein-Barr virus) or potentially KSHV or HHV-8 (Kaposi′s sarcoma Associated Herpes Virus or Human Herpes Virus 8) [4]. This one (HHV-8) (which one need to specify) was isolated from half of the patients. A disruption in the production of IL-6 resulting in lymphoproliferation B unregulated by a failed system is being advanced [5]. Both in the clinical and progressive plan, the angiomatous lymphoid hamartoma occurs in two forms, unicentricform (90% of cases) and multicentric form. The multicentric form was described first time by Gaba et al in 1978. Histologically, follicular hyperplasia is the predominant sign with mainly small polyclonal B lymphocytes with mantle cell phenotype CD5 (+). Clonal cytogenetic chromosomal abnormalities were found in the lymph nodes affected. Nakamura et al discloses a translocation [5] (p22; q22), which is related with the interleukin gene 6 (IL-6) [6].

The histological picture of the angiomatous lymphoid hamartoma is non pathognomonic and can occur in autoimmune diseases such as rheumatoid arthritis, primary Sjogren′s Syndrome Sjogren′s, scleroderma or lupus, in congenital or acquired immune deficiencies, in Kaposi′s sarcoma in the contiguous malignancies including lymphoma or in infectious diseases (HIV) [1, 7]. The differential diagnosis may also arise with POEMS syndrome. Peterson and Frizzera proposed four criteria for establishing the diagnosis of angiomatous lymphoid hamartoma multicenter: an evocative histopathology, often of plasma cell type, peripheral lymphadenopathy dominating the clinical setting, multiviceral affection, and the exclusion of another etiology [7].

The average age of patients with lymphoid angiomatoushamartoma multicenter is higher than those with unicentricform, with predominance in the sixth decade [7]. There is a slight male predominance with a sex ratio of 1.4. General symptoms (fever, night sweats, weight loss, and anorexia) are found in 95% of cases. The lymphadenopathy is present in all cases with multiple deep abdominal lymph nodes in 53% cases and mediastinal lymph nodes in 47% cases. Splenomegaly has been described in 68% of cases and hepatomegaly in 53% of cases. This patient (present case) was diagnosed with splenomegaly. Edema and pleural, pericardial effusion and ascites are found in half of the cases. The highly polymorphic skin lesions, is present in 55% of cases [1]. Our patient had a vascular purpura. Several cases of interstitial pneumonitis with infiltration by lymphocytes and plasma cells have been described [7]. Diarrhea and nausea are relatively frequent in these patients. Sensorimotor neuropathy and other events such as coma, aphasia, dysarthria or epilepsy have also been reported in these patients [1]. The rheumatic disorder is manifested by arthralgia, myalgia and joint effusions. Renal involvement, found in our patient, is varied with proteinuria, hematuria, nephrotic syndrome or renal insufficiency [1].

The association with systemic diseases is described in particular with scleroderma, primitive Gougerot-Sjogrensyndrome or mixed connectivity [1]. Laboratory abnormalities are varied but dominated by a typical inflammatory syndrome due to stimulation by IL-6 [1], accelerated rate of sedimentation, hypoalbuminénie, elevated transaminases and alkaline phosphatases. The polyclonal hypergammaglobulinemia, on IgG and IgA was found in 85% of cases [7]. A large number of autoantibodies are found, including antinuclear antibodies [8]. The bill of health done for our patient had isolated ANA flecked of a 1/100. Blood cell count [7] revealed anemia (89% of cases), leukopenia (21% of cases) or leukocytosis and thrombocytopenia (61%) in these patients. Four progressive forms of CD were mapped by Weisenburger et al [8]; aggressive and rapidly fatal form representing 24% of cases, as was the case in our patient who died in an array of renal failure; the chronic form, stable, little symptomatic, moving one piece on a large number of years, 16% of cases; the relapsing form, 40% of cases and, association with malignancy, 20% of cases. The prognosis of multicenteric angiomatous lymphoid hamartoma remains obscure, with a median survival of 30 months [7]; 26% of patients die within the first year after diagnosis and 13% are alive at 10 years and older. The disease progressionis aggressive in HIV (+) patients [9]. Death in CD is related to an infectious complication in 70% of cases or due to cancer in 30% of cases. Kaposi′s sarcoma [8], non-Hodgkin lymphoma, myeloma, disease Hodgkin and rarely carcinomas are main complications in CD. Laurens et al has reported that the risk of malignant transformation cannot be excluded in CD.

There are few data on the treatment of multicentric form of angiomatous lymphoid hamartoma because it shows the possibility of spontaneous remission, variability and evolution into difficult to assess response to the treatment retrospectively. Some advocate corticosteroids, while others radiation and/or chemotherapy while others recommend simply monitoring. Given the risk of progression, it is more logical to treat the forms with potentially serious approach. Using systemic corticosteroids prednisone at a dose of 1 to 2 mg/kg/ day should be maintained for several months and it can give a regression functional signs, and improved biological parameters. Immunosuppressants, including azathioprine and cyclophosphamide, have achieved a complete remission in combination with prednisone in some patients [9]. Chemotherapy using a single alkylating agent (chlorambucil, cyclophosphamide) or associated with Vincaalkaloides (CVP, COP, and CHOP) is indicated in cases that were resistant to corticoids. Interferon alpha has been tested in a plasma cell multicentric form with obtaining a complete answer, but a relapse occurred 11 months after stopping the treatment [9]. Radiation used in low doses (12 Gy) [10] resulted in a complete response in one case. The purine analogs may be a therapeutic alternative. Intravenous immunoglobulins have been successfully used by Crump et al, the patient was treated in complete remission with a decline of 13 months. The monoclonal anti-IL-6 is used in a patient with only a suspensive effect and could be a promising therapeutic perspective.

## Conclusion

The angiomatous lymphoid hamartoma is a rare illness. It seems to be secondary to the overproduction of interleukin-6 or role of HHV-8 is also incriminated. Systemic manifestations are always present and superficial lymph nodes are constant and varied. The association with Kaposi's sarcoma or lymphoma should be sought systematically. There is no therapeutic consensus, prognosis is guarded and death can occur even in the absence of malignant transformation. This justifies intensive therapy with high doses of corticosteroids or immunosuppressant's, when the disease is progressing rapidly.
